# Firefly Mating Algorithm for Continuous Optimization Problems

**DOI:** 10.1155/2017/8034573

**Published:** 2017-07-20

**Authors:** Amarita Ritthipakdee, Arit Thammano, Nol Premasathian, Duangjai Jitkongchuen

**Affiliations:** ^1^Computational Intelligence Laboratory, King Mongkut's Institute of Technology Ladkrabang, Bangkok 10520, Thailand; ^2^Faculty of Information Technology, King Mongkut's Institute of Technology Ladkrabang, Bangkok 10520, Thailand; ^3^College of Innovative Technology and Engineering, Dhurakij Pundit University, Bangkok, Thailand

## Abstract

This paper proposes a swarm intelligence algorithm, called firefly mating algorithm (FMA), for solving continuous optimization problems. FMA uses genetic algorithm as the core of the algorithm. The main feature of the algorithm is a novel mating pair selection method which is inspired by the following 2 mating behaviors of fireflies in nature: (i) the mutual attraction between males and females causes them to mate and (ii) fireflies of both sexes are of the multiple-mating type, mating with multiple opposite sex partners. A female continues mating until her spermatheca becomes full, and, in the same vein, a male can provide sperms for several females until his sperm reservoir is depleted. This new feature enhances the global convergence capability of the algorithm. The performance of FMA was tested with 20 benchmark functions (sixteen 30-dimensional functions and four 2-dimensional ones) against FA, ALC-PSO, COA, MCPSO, LWGSODE, MPSODDS, DFOA, SHPSOS, LSA, MPDPGA, DE, and GABC algorithms. The experimental results showed that the success rates of our proposed algorithm with these functions were higher than those of other algorithms and the proposed algorithm also required fewer numbers of iterations to reach the global optima.

## 1. Introduction

Optimization is a process of finding the most desirable solution to the problem of interest. Optimization problems can be divided into two main categories depending on the types of variables involved (discrete or continuous). A problem with discrete variables is known as a combinatorial optimization problem while a problem with continuous variables is known as a continuous optimization problem. The optimization algorithm proposed in this paper only emphasizes solving continuous optimization problems. Optimization algorithms are of many varieties ranging from simple search to much more complex ones that require a great deal of computational time. At present, among the widely researched types of optimization algorithms, self-organized algorithms employing procedures that emulate animal swarm behavior are very popular. Brief overviews of recent metaheuristic algorithms based on firefly, ant, fish, bird, and bee are presented in the next paragraph.

Yang [1] developed a swarm intelligent algorithm based on the flashing behavior of fireflies. In nature, the firefly flashes its light to attract other fireflies for mating. The less bright firefly is attracted and moves towards the one that flashes brighter light. The experimental results in Yang's paper showed that the firefly algorithm outperformed both genetic algorithm and particle swarm optimization on the tested benchmarks. Similar results were also obtained by Baykasoğlu and Ozsoydan [2]. Even though the algorithm is very promising, further investigation is needed to improve on the convergence of the algorithm and to prevent trapping in local optima. Farahani et al. [3] proposed an improved firefly algorithm called Gaussian distribution firefly (GD-FF) algorithm. Three new strategies were introduced: (i) an adaptive step length that changes with time, (ii) a directed movement towards the global best solution when there is no brighter firefly within its vicinity, and (iii) a social behavior that changes the position of each firefly based on a Gaussian distribution. The authors found that their algorithm got better result and converged to the global optimum faster than the original firefly algorithm. Rizk-Allah et al. [4], on the other hand, improved the firefly algorithm in a different way; they combined it with an ant colony optimization (ACO) algorithm. First, this hybrid algorithm is initialized using the ACO algorithm. Then the firefly algorithm is put to work as a local search to refine the solutions found by the ants. The hybrid was proven successful. Shin and Kita [5] improved the performance of particle swarm optimization (PSO) by including the information of the second global best and second personal best particles in their developed algorithm. The algorithm's performance was found to be better than the original PSO. Another variant of PSO was an algorithm called Levy flight particle swarm optimization (LFPSO) [6]. This algorithm combined PSO with Levy flight. Experimental results showed that the LFPSO clearly outperformed the original PSO and other PSO variants in terms of solution quality and robustness. A different kind of swarm intelligent algorithm based on the behavior of bees in finding food sources, called bee colony optimization (BCO), was also further developed by assigning two weights to each food source. One is a global weight and the other is a local weight. As a result, the proposed algorithm, named weighted bee colony optimization (wBCO) [7], performed better than the original BCO and many state-of-the-art algorithms. Artificial bee colony (ABC), another bee-inspired algorithm, was modified by Gao and Liu [8]. In their proposed algorithm, Levy flight, differential evolution, and particle swarm optimization were incorporated into the original ABC algorithm. Experimental results showed that the proposed algorithm achieved better performance than the original ABC algorithm and many of its variants. Niu et al. [9] proposed an improved version of the original fruit fly optimization algorithm (FOA), called DFOA. They modified the expression of the smell concentration judgement value and introduced DE/best/1 mutation strategy to replace the random search. The results showed that DFOA was superior to seven other evolutionary computation methods. Chen et al. [10] presented a new PSO variant called particle swarm optimization with an aging leader and challengers (ALC-PSO). In ALC-PSO, the leader of the swarm has a lifespan. If the leader is trapped in a local optimum, it will age quickly and new challengers will emerge to replace the old leader. As a result, ALC-PSO has an ability to escape from local optima and to find global optima in a short period of time. Genetic algorithm (GA) was also developed further by Thakur [11] who proposed a new crossover operator, called double Pareto crossover operator (DPX). DPX was used in combination with power mutation (PM) operator. He tested this algorithm with multimodal nonlinear functions and found that its success rate was higher than that of the standard GA.

In this paper, a swarm intelligence algorithm called firefly mating algorithm (FMA) is presented. In FMA, GA is used as the core of the algorithm. A new mating pair selection method, inspired by the mating behavior of fireflies in nature, is introduced and incorporated into GA.

The rest of the paper is organized as follows: Sections 2 and 3 briefly discuss the original firefly algorithm and genetic algorithm, respectively; in Section 4, the biological background of the proposed algorithm is explained; in Section 5, the proposed model with a detailed description of each step is presented; in Section 6, a description of the experiments is given; in Section 7, experimental results are shown and compared; finally, Section 8 is the conclusion.

## 2. Firefly Algorithm

This section describes the original firefly algorithm. Firefly algorithm (FA) is a reliable and efficient metaheuristic algorithm capable of solving many real-world problems such as scheduling, optimization problems in dynamic environments, and economic load dispatch problem. This algorithm is influenced by the flashing behavior of fireflies to attract one another. It is constructed based on three rules [12]:

(i) All fireflies are unisex so that one firefly is attracted to all other fireflies.

(ii) The attractiveness of a firefly is proportional to its brightness. For any two fireflies, the dimmer one is attracted by (and thus moves towards) the brighter one. However, if there are no fireflies brighter than a given firefly, that firefly will move randomly.

(iii) The brightness of a firefly decreases as the distance from it increases. This is because light is absorbed when it passes through the medium. Therefore, the brightness (also attractiveness) of the firefly *j* seen by the firefly *i* is defined in (1).

(1) βjr=βj0e−γr2,

(2) r=xi−xj=∑k=1dxik−xjk2,

where *γ* is a light absorption coefficient of the medium, *r* is the Euclidean distance between the firefly *i* and the firefly *j*, *β*_*j*_(0) is the brightness of the firefly *j* at *r* = 0, and *x*_*i*_ and *x*_*j*_ are the locations of the fireflies *i* and *j*, respectively.

If the firefly *j* is the brighter one, the value of its attractiveness regulates the movement of the firefly *i* according to the following equation:

(3) $$\begin{array}{c} x_{i}=x_{i}+\left[\;\beta_{j}\left(\;r\;\right)\;\right]\left(\;x_{j}-x_{i}\;\right)+\alpha \left(\;rand\;\right), \end{array}$$

where *α* is a randomization parameter and rand is a uniform random number in the range [−0.5,0.5]. The function of the second term in (3) is to move the firefly *i* towards the firefly *j*. The function of the third term in (3) is to move the solution away from a local optimum when such incident takes place.

## 3. Genetic Algorithm

Genetic algorithm (GA) is an optimization technique inspired by the process of natural selection. GA starts with an initial population which consists of a number of randomly generated chromosomes. A new population is created from the current one by means of 3 genetic operators: selection, crossover, and mutation. The selection operator stochastically chooses chromosomes to be included in the mating pool; the ones with higher fitness values are more likely to be chosen. Crossover operator selectively chooses some genes from the chromosomes of the parents and combines them into the offspring. Mutation operator randomly changes some genes of the offspring. This evolution process is repeatedly performed until any of the stopping criteria is met. The commonly used stopping criteria are as follows: (i) a predefined number of iterations is reached, (ii) the best solution does not improve for a predefined number of iterations, and (iii) a large percentage of the chromosomes in the population is the same.

## 4. Biological Inspiration of the Proposed Algorithm

There are over 2,000 species of fireflies around the world, but most of them are found in the tropical zone. They live under the water when they are larvae and on the ground and in the air when they are adults. Fireflies are social animals. They stay in a swarm on tree branches and lay eggs on the ground around the trees. The fascinating thing when observing fireflies is their light flash. The light emitted from their abdomens is a cold light through chemical reactions within their bodies. Fireflies emit flashing light for communication, luring preys, repelling predators, and attracting mates. Note that this research only focuses on the mating behavior of the firefly; therefore, the detailed relation of the flashing light signal to the mate selection process is further discussed in the next paragraph.

In the mating season, female fireflies release pheromones into the air to signal their readiness to mate. The pheromones are carried away in the direction controlled by the wind [13]. Male fireflies follow the pheromone trail and approach the females from a downwind direction [13]. More males are attracted to females who release more pheromones. Males then fly around the trees that the females perch on and flash courtship signal to attract females [14]. Females are more attracted to brighter males and response to those males by flashing their own lights. Then many rounds of mating take place during the night [15]. Males mate until they run out of sperm in their sperm reservoir while females can hold only a certain amount of sperms in their spermatheca. Sperms from fitter males are more likely to be chosen to fertilize a female's eggs; this is due to the following two explanations: (i) females have ways to discard low-quality sperms, including destroying them by their internally produced chemicals [16, 17]; (ii) one male's seminal fluid can incapacitate rival males' sperm within the female reproductive tract [16, 18]. It is nature's way of selective breeding.

## 5. Our Proposed Algorithm

This section describes a firefly mating algorithm (FMA), which is built on top of the GA. FMA incorporates a new mating feature, inspired by the mating behavior of fireflies in nature, into GA. This new feature, which will be discussed in Section 5.2, significantly contributes to the capability of the proposed FMA.

FMA consists of three main processes: (i) a male selects a female according to the level of her released pheromone that he senses which changes according to wind speed and direction; (ii) a female selects a male according the light intensity of his flash; and (iii) a male or a female mates repeatedly until he runs out of sperms or her spermatheca is full, producing more able offspring for the next generation. This algorithm proceeds from the first step to the last in the 6 following steps.

### 5.1. Initialization

An initial population of *N* fireflies is randomly generated; half are assigned as males and the other half are females. Each firefly consists of *d* genes (which is equal to the number of variables in the problem). Additionally, the sizes of each female's spermatheca and each male's sperm reservoir, which are real numbers between 0 and 1, are randomly generated. Lastly, the fitness value of each firefly is calculated.

### 5.2. Selection of Mating Pairs

This step features a new method for determining the mating pairs. Unlike in the GA where mating pairs are selected by using a roulette wheel selection technique, our proposed algorithm introduces a new selection method based on a process of firefly mate selection. First, male fireflies are drawn to a female's location by following the pheromone that the female releases. Second, the female selects males based on their brightness. Finally, mating pairs are formed based on the mutual attraction of each pair. By introducing this concept to the algorithm, it provides a way to overcome the problem of getting stuck in local optima, as is often the case with the roulette wheel selection technique. A detail of this new selection method is described in the following subsection.

#### 5.2.1. Determination of Female Sex Appeal

Female sex appeal is directly proportional to the amount of pheromone released by the female. The pheromone released by the female firefly diffuses downwind and reaches each male firefly in unequal amounts depending on 2 factors: (i) the distance and (ii) the speed and direction of the wind. In this subsection, therefore, the male fireflies determine the concentration level of each female's pheromone reaching them. A highly fit female that is farther away from a male can get selected by the male if her highly concentrated pheromone gets carried along by a high-speed wind towards the male. The concentration level of the female *i*'s pheromone reaching the male *j*, *P*_*ji*_, is calculated by using

(4) $$\begin{array}{c} P_{ji}=f_{i}\times \left(\;\vec{D}\cdot \vec{W}\;\right), \end{array}$$

where *f*_*i*_ is the fitness value of a female firefly *i*, $\vec{W}$ is the wind vector which is randomly generated in each iteration, and $\vec{D}$ is the difference vector between the position vector of a male and that of the female.

#### 5.2.2. Determination of Male Sex Appeal

In this subsection, the female fireflies determine the appeal of each male in their vicinity. Similar to the original FA, the appeal of a male firefly is directly proportional to his brightness. Thus, by imitating (1), the appeal of the male firefly *j* seen by the female firefly *i* is calculated according to the following equation:

(5) $$\begin{array}{c} A_{ij}=f_{j}e^{-\gamma r^{2}}, \end{array}$$

where *A*_*ij*_ is the appeal of the male firefly *j* seen by the female firefly *i*, *γ* is a constant in the range [0,1], *r* is the Euclidean distance between the male and the female (calculated according to (2)), and *f*_*j*_ is the fitness value of the male firefly *j*.

#### 5.2.3. Calculation of the Mutual Attraction

According to the degree of mutual attraction, a number of males and females are paired together as potential parents. A pair whose mutual attraction value is the highest among all pairs is selected as the first mating pair. The mutual attraction between the female firefly *i* and the male firefly *j*, MA_*ij*_, is defined as

(6) $$\begin{array}{c} MA_{ij}=A_{ij}+P_{ji}. \end{array}$$

After each mating, the numbers of sperms in the male's sperm reservoir and in the female's spermatheca are updated. The update procedure is described in the next subsection. Next, the pair with the next highest value is selected if its male member still has some sperms left in the sperm reservoir and the female's sperm bucket has not become full yet.

#### 5.2.4. Update Procedure for Male's Sperm Reservoir and Female's Spermatheca

At the beginning, the number of sperms in each male's sperm reservoir and the size of each female's spermatheca are randomly initialized within the range [0,1]. When a pair of male and female mates, sperms are transferred from the male's sperm reservoir to the female's spermatheca, and the number of sperms in the male's sperm reservoir is reduced. For each mating, the number of sperms a male gives to the female he chose depends on her fitness. The number of sperms that a male can transfer to the female he chose is calculated by the following equation:

(7) $$\begin{array}{c} \eta_{ij}=\delta_{j}\times f_{i}, \end{array}$$

where *η*_*ij*_ is the number of sperms transferred to the female at the time of mating, *δ*_*j*_ is the number of sperms in the sperm reservoir, and *f*_*i*_ is the fitness value of a female firefly *i*. It stands to reason that a fitter female should get more sperms because, then, she would produce more offspring that are fitter than those produced by other females.

After mating, the number of sperms in each male's sperm reservoir and the space of each female's spermatheca are checked. Once a male runs out of sperm or a female's spermatheca is full, he/she will be disqualified from mating in the next round. Equations (8) and (9) are used to update the number of sperms in a male's sperm reservoir and a female's spermatheca, respectively.

(8) δjnew=δjold−ηij,

(9) ωinew=ωiold+ηij.

Mating goes on until there is no qualified firefly left to form the mating pair.

### 5.3. Crossover Operation

When a male and a female mates, some of their genes are crossed over to form two new offspring. One of the two following cases is applied with a specific crossover procedure as follows.

**Case 1 .**

**Figure 1:**
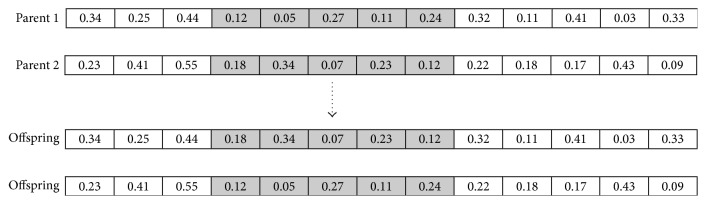
Example of 2-point crossover.

If the parents have never been mated before, the 2-point crossover operator (Figure 1) is used to create the offspring. The 2-point crossover operator starts from randomly selecting a start position and an end position in the parent chromosomes to be crossed over, and then the genes between these two positions are crossed over and placed in the same position in the chromosomes of the two offspring, each having the original genes from each parent before the crossover.

**Case 2 .**

**Figure 2:**
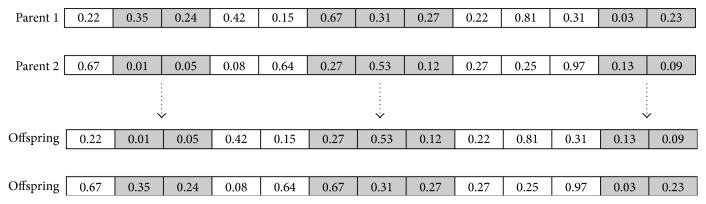
Example of *n*-point crossover.

If either of the parents has been mated before, the *n*-point crossover operator (Figure 2) is used to create the offspring. The *n*-point crossover operator starts from randomly selecting *n*/2 pairs of back-to-back start and end positions, then crossing over the genes from the parents between each pair of start and end position. The rest of the genes are retained.

### 5.4. Mutation Operation

**Figure 3:**
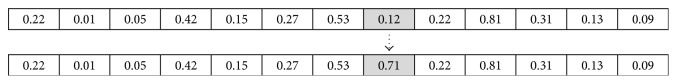
Example of the mutation operation.

After an incipient offspring is produced from mating, some of its genes are randomly changed (mutated) to new values within the range of the variables as shown in Figure 3. The mutation is performed in order to promote diversity of the population and to help avoid getting stuck in local optima.

### 5.5. Selection of the Population for the Next Generation

After all offspring are mutated, the *N* best fireflies out of the combined population of parents and offspring are selected to replace the old population of parents; in effect, only the more effective fireflies are selected to be the population of the next generation. The selection is done by sorting members by their fitness values and then selecting only the members with the higher fitness values that make up the total number of members of the initial population.

### 5.6. Termination

After the selection of a new population for the next generation, the current iteration is completed. The algorithm then moves on to perform the next iteration until the specified maximum number of iterations is reached or the best solution does not improve for a predefined number of iterations.

## 6. Description of the Experiments

Our FMA algorithm was performance-tested on Core-i7 computers, and the test results were compared against those of firefly algorithm (FA) and 11 other widely cited algorithms in the literature, namely, ALC-PSO [10], COA [19], MCPSO [20], LWGSODE [21], MPSODDS [22], DFOA [9], SHPSOS [23], LSA [24], MPDPGA [25], DE [26], and GABC [27]. These algorithms were chosen as benchmarks because they are variants of the prominent bioinspired algorithms such as particle swarm optimization, genetic algorithm, differential evolution algorithm, and artificial bee colony algorithm. Moreover, from the literature, their performance was superior to that of their original counterparts. Twenty well-known standard test functions (*f*_1_–*f*_20_) were employed to test whether these algorithms would be able to solve all common types of problems. In particular, *f*_1_–*f*_16_ are high-dimensional (30 dimensions) that represent computationally complex tasks while *f*_17_–*f*_20_ are two-dimensional multimodal functions. The descriptions of the test functions are shown in Table 1. Table 1 provides the following information about each test function: function ID, equation, dimension (*d*), search domain (*S*), and optimum value (*f*_min_). For each test function, five experimental repetitions were performed with different initial populations each time. Since the performance of the metaheuristic algorithms strongly depends on proper selection of system parameters, as most of other intelligent systems, the parameters of FMA are varied to a wide extent in order to get the best out of the algorithm. As a result, the best value for each parameter is shown in Table 2. The performance of FMA was tested in terms of the following 3 aspects: (i) the best solution that an algorithm found, (ii) the average number of iterations to reach the global optimum (in the case that an algorithm has found it), and (iii) the success rate or the number of times that an algorithm was able to find the global optimum divided by the total number of trials.

## 7. Results and Discussion

The performance of all compared algorithms in finding the optimal solutions of 20 test functions is shown in Table 3. The number shown in each cell of the table is the best result obtained by each algorithm. The symbol “—” denotes that the algorithm was not tested on that function. Compared to other algorithms, FMA surpassed them all with respect to the percentage of functions in which the algorithm successfully found the global optima. FMA was able to find the global optima in 16 out of 20 functions tested (shown in bold typeface in Table 3). FA found the global optima of 9 out of 20 test functions. It failed to find the global optima of test functions *f*_3_, *f*_4_, *f*_6_, *f*_8_, *f*_10_, *f*_12_, *f*_13_, *f*_14_, *f*_15_, *f*_17_, and *f*_18_. ALC-PSO found the global optima of test functions *f*_6_, *f*_8_, and *f*_10_ but failed to find the global optima of test functions *f*_1_, *f*_2_, *f*_5_, *f*_7_, *f*_9_, and *f*_13_. COA found the global optima of test functions *f*_1_, *f*_2_, *f*_6_, *f*_7_, *f*_10_, and *f*_20_ but failed to find the global optima of test functions *f*_4_, *f*_5_, *f*_9_, *f*_12_, *f*_13_, and *f*_19_. MCPSO found the global optima of test functions *f*_1_ and *f*_2_ but failed to find the global optima of test functions *f*_5_, *f*_7_, *f*_9_, *f*_10_, *f*_12_, and *f*_18_. LWGSODE and MPSODDS were not able to locate the global optima in any of the functions tested. DFOA successfully found the global optimum of only 1 out of 10 functions tested. SHPSOS found the global optima of test functions *f*_1_, *f*_2_, *f*_3_, *f*_6_, *f*_7_, *f*_8_, and *f*_16_ but failed to find the global optima of test functions *f*_4_, *f*_9_, *f*_10_, *f*_12_, *f*_13_, *f*_17_, and *f*_18_. LSA found the global optima of test functions *f*_6_ and *f*_20_ but failed to find the global optima of test functions *f*_1_, *f*_2_, *f*_4_, *f*_5_, *f*_7_, *f*_9_, *f*_10_, *f*_12_, *f*_13_, and *f*_19_. MPDPGA found the global optima of test functions *f*_1_, *f*_7_, and *f*_11_ but failed to find the global optima of test functions *f*_5_, *f*_9_, and *f*_10_. DE found the global optima of test functions *f*_1_, *f*_2_, *f*_5_, *f*_6_, *f*_7_, *f*_10_, and *f*_20_ but failed to find the global optima of test functions *f*_4_, *f*_9_, *f*_12_, and *f*_13_. DFOA successfully found the global optimum of only 1 out of 9 functions tested.

Besides being very effective, FMA also has good convergence performance.  Table 4 shows the comparative convergence performance of FMA and FA on the following 2 aspects: the average number of iterations to reach the global optimum (in the case that an algorithm has found it) and the success rate (the number of times that an algorithm was able to find the global optimum divided by the total number of trials). “N/A” denotes that the algorithm was unable to find the optimal solution for that function. By examining the results, the following are summaries of the finding. First, FMA achieved 100% success rate for 15 out of 16 functions in which FMA found the global optima. This means that FMA almost always found the global optimum regardless of the difference in the initial population of each experimental trial. Second, as mentioned earlier, FMA was able to find the global optima in 16 out of 20 functions while FA only found 9. Therefore, only eight functions (*f*_1_, *f*_2_, *f*_5_, *f*_7_, *f*_9_, *f*_11_, *f*_16_, and *f*_20_) in which both FMA and FA found the global optima have been analyzed. Out of the 8 functions, FMA can achieve the global optima with far fewer iterations than FA for the functions *f*_2_, *f*_7_, *f*_9_, *f*_11_, *f*_16_, and *f*_20_, while FA has better convergence rate for the functions *f*_1_ and *f*_5_.

The distinct advantage of FMA is the rich diversity of offspring that it generates. Two main causes for this kind of diversity are that FMA enables fireflies to perform multiple mating and that the level of female pheromone that a male senses depends not only on the distance between them but also on the wind speed and direction which is randomly selected for each iteration. As a result, FMA is better able to converge to a global optimum of a function that has a wide and complex search space.

## 8. Conclusion

In this paper, a new mating pair selection technique, inspired by the mating behavior of fireflies in nature, is proposed and incorporated into the original firefly algorithm and genetic algorithm. The performance of FMA was tested with sixteen 30-dimensional benchmark functions and four 2-dimensional functions against several widely accepted algorithms. The test results showed that it was able to converge to the global optima in 16 of them. Moreover, compared to the firefly algorithm, FMA required fewer numbers of iterations for most of the functions tested. Because of this initial success, FMA is worth further investigation and application to combinatorial optimization problems.

## Figures and Tables

**Table 1:** Test functions.

Function ID	Equation	*d*	*S*	*f*_min_
*f*_1_	$f\left(x\right)=\overset{d}{\underset{i=1}{\sum }}x_{i}^{2}$	30	[−100,100]^*d*^	0

*f*_2_	$f\left(x\right)=\overset{d}{\underset{i=1}{\sum }}\left|x_{i}\right|+\overset{d}{\underset{i=1}{\prod }}\left|x_{i}\right|$	30	[−10,10]^*d*^	0

*f*_3_	$f\left(x\right)=\overset{d}{\underset{i=1}{\sum }}ix_{i}^{4}$	30	[−1.28,1.28]^*d*^	0

*f*_4_	$f\left(x\right)=\overset{d}{\underset{i=1}{\sum }}ix_{i}^{4}+random\left(0,1\right)$	30	[−1.28,1.28]^*d*^	0

*f*_5_	$f\left(x\right)=\overset{d-1}{\underset{i=1}{\sum }}\left[100{\left(x_{i+1}-x_{i}^{2}\right)}^{2}+{\left(x_{i}-1\right)}^{2}\right]$	30	[−30,30]^*d*^	0

*f*_6_	$f\left(x\right)=\overset{d}{\underset{i=1}{\sum }}{\left(\left[x_{i}+0.5\right]\right)}^{2}$	30	[−100,100]^*d*^	0

*f*_7_	$f\left(x\right)=10d+\overset{d}{\underset{i=1}{\sum }}\left[x_{i}^{2}-10\cos\left(2\pi x_{i}\right)\right]$	30	[−5.12, 5.12]^*d*^	0

*f*_8_	$f\left(x\right)=\overset{d}{\underset{i=1}{\sum }}\left[y_{i}^{2}-10\cos\left(2\pi y_{i}\right)+10\right]$, $y_{i}=\left\{\begin{array}{cc} x_{i}, & \left|x_{i}\right|<0.5\\ \frac{round\left(2x_{i}\right)}{2}, & \left|x_{i}\right|\geq 0.5 \end{array}\right.$	30	[−5.12,5.12]^*d*^	0

*f*_9_	$f\left(x\right)=-20\exp\left(-0.2\sqrt{\frac{1}{d}\overset{d}{\underset{i=1}{\sum }}x_{i}^{2}}\right)-\exp\left(\frac{1}{d}\overset{d}{\underset{i=1}{\sum }}\cos\left(2\pi x_{i}\right)\right)+20+\exp\left(1\right)$	30	[−32.768,32.768]^*d*^	0

*f*_10_	$f\left(x\right)=\frac{1}{4000}\overset{d}{\underset{i=1}{\sum }}x_{i}^{2}-\overset{d}{\underset{i=1}{\prod }}\cos\left(\frac{x_{i}}{\sqrt{i}}\right)\;+1$	30	[−600, 600]^*d*^	0

*f*_11_	$f\left(x\right)=\overset{d}{\underset{i=1}{\sum }}{\left|x_{i}\right|}^{i+1}$	30	[−1,1]^*d*^	0

*f*_12_	$f\left(x\right)=\frac{\pi }{d}\left\{10\;\sin^{2}\left(\pi y_{1}\right)+\overset{d-1}{\underset{i=1}{\sum }}{\left(y_{i}-1\right)}^{2}\left[1+10\;\sin^{2}\left(\pi y_{i+1}\right)\right]+{\left(y_{d}-1\right)}^{2}\right\}+\overset{d}{\underset{i=1}{\sum }}u\left(x_{i},10,100,4\right)$, $y_{i}=1+\frac{1}{4}\left(x_{i}+1\right)$, $u\left(x_{i},a,k,m\right)=\left\{\begin{array}{cc} k{\left(x_{i}-a\right)}^{m}; & x_{i}>a\\ 0; & -a\leq x_{i}\leq a\\ k{\left(-x_{i}-a\right)}^{m}; & x_{i}<-a \end{array}\right.$	30	[−50,50]^*d*^	0

*f*_13_	$f\left(x\right)=0.1\left\{sin^{2}\left(3\pi x_{i}\right)+\overset{d}{\underset{i=1}{\sum }}{\left(x_{i}-1\right)}^{2}\left[1+\sin^{2}\left(3\pi x_{i}\right)\right]+{\left(x_{d}-1\right)}^{2}\left[1+sin^{2}\left(2\pi x_{d}\right)\right]\right\}+\overset{d}{\underset{i=1}{\sum }}u\left(x_{i},5,100,4\right)$	30	[−50,50]^*d*^	0

*f*_14_	$f\left(x\right)=\overset{d/4}{\underset{i=1}{\sum }}\left[{\left(x_{4i-3}+10x_{4i-2}\right)}^{2}+5{\left(x_{4i-1}-x_{4i-2}\right)}^{2}+{\left(x_{4i-2}-2x_{4i-1}\right)}^{4}+10{\left(x_{4i-3}-x_{4i}\right)}^{4}\right]$	30	[−4,5]^*d*^	0

*f*_15_	$f\left(x\right)=\sin^{2}\left(\pi w_{1}\right)+\overset{d-1}{\underset{i=1}{\sum }}\left\{{\left(w_{i}-1\right)}^{2}\left[1+10\sin{\left(\pi w_{i}+1\right)}^{2}\right]\right\}+{\left(w_{n}-1\right)}^{2}{\left[1+\sin\left(2\pi w_{d}\right)\right]}^{2}$, $w_{i}=1+\frac{x_{i}-1}{4}$	30	[−10, 10]^*d*^	0

*f*_16_	$f\left(x\right)=\overset{d}{\underset{i=1}{\sum }}ix_{i}^{2}$	30	[−10,10]^*d*^	0

*f*_17_	$f\left(x\right)=0.5+\frac{\sin^{2}\sqrt{\sum_{i=1}^{d}x_{i}^{2}}-0.5}{{\left(1+0.001\sum_{i=1}^{d}x_{i}^{2}\right)}^{2}}$	2	[−100,100]^*d*^	0

*f*_18_	$f\left(x\right)=418.9829d-\overset{d}{\underset{i=1}{\sum }}\left(x_{i}\sin\sqrt{\left|x_{i}\right|}\right)$	2	[−500,500]^*d*^	0

*f*_19_	$f\left(x\right)=4x_{1}^{2}-2.1x_{1}^{4}+\frac{1}{3}x_{1}^{6}+x_{1}x_{2}-4x_{2}^{2}+4x_{2}^{4}$	2	[−5,5]^*d*^	−1.03163

*f*_20_	*f*(*x*) = [1 + (*x*_1_ + *x*_2_ + 1)^2^(19 − 14*x*_1_ + 3*x*_1_^2^ − 14*x*_2_ + 6*x*_1_*x*_2_ + 3*x*_2_^2^)] · [30 + (2*x*_1_ − 3*x*_2_)^2^(18 − 32*x*_1_ − 12*x*_1_^2^ + 48*x*_2_ − 36*x*_1_*x*_2_ + 27*x*_2_^2^)]	2	[−2,2]^*d*^	3

**Table 2:** The parameter setting.

Parameters	Values
Population size	300
Maximum number of iterations	5000
Light absorption	0.2
Crossover rate	1.0
Mutation rate	0.2

**Table 3:** Performance comparison between FMA and other algorithms.

	FMA	FA	ALC-PSO	COA	MCPSO	LWGSODE	MPSODDS	DFOA	SHPSOS	LSA	MPDPGA	DE	GABC
*f*_1_	0	0	1.135*E* − 172	0	0	1.13*E* − 8	1.579*E* − 69	3.09*E* − 7	0	1.0622*E* − 19	0	0	3.8838*E* − 17
*f*_2_	0	0	1.121*E* − 98	0	0	2.78*E* − 4	1.96*E* − 42	5.13*E* − 3	0	2.21758*E* − 7	—	0	—
*f*_3_	0	4.64*E* − 7	—	—	—	1.34*E* − 16	—	—	0	—	—	—	—
*f*_4_	0.36400	0.0113	—	3.4479*E* − 5	—	—	—	—	4.58*E* − 4	0.016268885	—	7.73*E* − 7	—
*f*_5_	0	0	3.729*E* − 7	4.1238*E* − 4	6.12	2.66*E* + 1	0.604493	1.76*E* − 5	—	0.560036507	0.00612658	0	1.7632*E* − 3
*f*_6_	0	1.57*E* − 5	0	0	—	—	—	0	0	0	—	0	—
*f*_7_	0	0	7.105*E* − 15	0	7.08*E* − 6	8.77*E* − 7	187.44934	6.95*E* − 7	0	40.7932709	0	0	0
*f*_8_	0.25200	1.04*E* − 9	0	—	—	—	—	0.0013	0	—	—	—	—
*f*_9_	0	0	7.694*E* − 15	8.8818*E* − 16	6.38*E* − 12	6.82*E* − 5	2.23*E* − 14	7.99*E* − 15	4.44*E* − 15	8.73041*E* − 8	0.00395865	4.44*E* − 16	7.9936*E* − 15
*f*_10_	0	1.38*E* − 3	0	0	1.91*E* − 14	1.44*E* − 10	0.0275372	6.69*E* − 6	5.84*E* − 4	2.22045*E* − 16	0.05830217	0	1.1102*E* − 16
*f*_11_	0	0	—	—	—	—	—	—	—	—	0	—	—
*f*_12_	0	2.95*E* − 4	—	1.5705*E* − 32	2.54*E* − 8	—	0.0540318	3.34*E* − 8	2.64*E* − 9	2.23008*E* − 15	—	1.57*E* − 32	—
*f*_13_	0	0.0448	1.350*E* − 31	1.3498*E* − 32	—	—	—	8.2*E* − 9	4.28*E* − 8	2.75229*E* − 19	—	1.3*E* − 32	—
*f*_14_	7.58*E* − 10	1.85*E* − 10	—	—	—	—	—	—	—	—	—	—	3.3927*E* − 5
*f*_15_	0	0.0144	—	—	—	—	—	—	—	—	—	—	5.5574*E* − 17
*f*_16_	0	0	—	—	—	—	—	—	0	—	—	—	4.8502*E* − 17
*f*_17_	0	0.0907	—	—	—	—	—	—	4.88*E* − 3	—	—	—	—
*f*_18_	0	0.0214	—	—	1.32*E* − 3	—	—	—	2.52*E* − 26	—	—	—	1.9843*E* − 4
*f*_19_	−1.03124	−1.03163	—	−1.0316	—	—	—	—	—	−1.031628453	—	—	—
*f*_20_	3	3	—	3	—	—	—	—	—	3	—	3	—

**Table 4:** Success rates and average numbers of iterations achieved by FMA and FA.

	FMA		FA	
	Average number of iterations	Success rate	Average number of iterations	Success rate
*f*_1_	126.8	100%	47.2	100%
*f*_2_	51.6	100%	229.6	100%
*f*_3_	32	100%	N/A	0%
*f*_4_	N/A	0%	N/A	0%
*f*_5_	214.4	100%	83	100%
*f*_6_	158	100%	N/A	0%
*f*_7_	84.2	100%	120	100%
*f*_8_	N/A	0%	N/A	0%
*f*_9_	62.8	100%	207.8	100%
*f*_10_	90.8	100%	N/A	0%
*f*_11_	80	100%	162	100%
*f*_12_	133.8	100%	N/A	0%
*f*_13_	101.5	40%	N/A	0%
*f*_14_	N/A	0%	N/A	0%
*f*_15_	113	100%	N/A	0%
*f*_16_	102.4	100%	284	100%
*f*_17_	280.6	100%	N/A	0%
*f*_18_	171	100%	N/A	0%
*f*_19_	N/A	0%	64.2	100%
*f*_20_	67	100%	125.2	100%
